# The Effect of Two Speed Endurance Training Regimes on Performance of Soccer Players

**DOI:** 10.1371/journal.pone.0138096

**Published:** 2015-09-22

**Authors:** F. Marcello Iaia, Matteo Fiorenza, Enrico Perri, Giampietro Alberti, Grégoire P. Millet, Jens Bangsbo

**Affiliations:** 1 Department of Biomedical Sciences for Health, Università degli Studi di Milano, Milan, Italy; 2 Department of Nutrition, Exercise and Sports, University of Copenhagen, Copenhagen, Denmark; 3 ISSUL, Institute of Sport Sciences, University of Lausanne, Lausanne, Switzerland; Norwegian University of Science and Technology, NORWAY

## Abstract

In order to better understand the specificity of training adaptations, we compared the effects of two different anaerobic training regimes on various types of soccer-related exercise performances. During the last 3 weeks of the competitive season, thirteen young male professional soccer players (age 18.5±1 yr, height 179.5±6.5 cm, body mass 74.3±6.5 kg) reduced the training volume by ~20% and replaced their habitual fitness conditioning work with either speed endurance production (SEP; n = 6) or speed endurance maintenance (SEM; n = 7) training, three times per wk. SEP training consisted of 6–8 reps of 20-s all-out running bouts followed by 2 min of passive recovery, whereas SEM training was characterized by 6–8 x 20-s all-out efforts interspersed with 40 s of passive recovery. SEP training reduced (p<0.01) the total time in a repeated sprint ability test (RSA_t_) by 2.5%. SEM training improved the 200-m sprint performance (from 26.59±0.70 to 26.02±0.62 s, p<0.01) and had a *likely* beneficial impact on the percentage decrement score of the RSA test (from 4.07±1.28 to 3.55±1.01%) but induced a *very likely* impairment in RSA_t_ (from 83.81±2.37 to 84.65±2.27 s). The distance covered in the Yo-Yo Intermittent Recovery test level 2 was 10.1% (p<0.001) and 3.8% (p<0.05) higher after SEP and SEM training, respectively, with *possibly* greater improvements following SEP compared to SEM. No differences were observed in the 20- and 40-m sprint performances. In conclusion, these two training strategies target different determinants of soccer-related physical performance. SEP improved repeated sprint and high-intensity intermittent exercise performance, whereas SEM increased muscles’ ability to maximize fatigue tolerance and maintain speed development during both repeated all-out and continuous short-duration maximal exercises. These results provide new insight into the precise nature of a stimulus necessary to improve specific types of athletic performance in trained young soccer players.

## Introduction

A challenge in sport physiology is how to improve athletic performance.

In recent years, exercise characterized by short duration (10–30 s) maximal/near maximal bursts, also termed speed endurance training, has emerged as an innovative and time efficient strategy to induce rapid physiological remodelling and enhance work capacity [1].

The rising advances in biotechnologies have also provided additional knowledge on the signaling mechanisms underpinning exercise-induced skeletal muscle adaptation. Particularly, it was revealed how different training interventions promote the transcription of selected genes relevant for enhancing the physiological systems that limit specific athletic performances [2,3]. Thus, genetic and molecular responses to exercise, training adaptations and thereafter performance improvements appear to be highly specific to the stimulus applied (i.e. exercise mode, intensity, volume and frequency) [2,3].

However, it remains unclear which peculiar features of a training stimulus are required to induce optimal adaptations in targeted areas. A potential key element for determining the adaptive response is the duration of the recovery intervals, or the exercise:recovery ratio [4,5]. Therefore, the first step for studying such processes would be to select anaerobic training regimes differing in work/rest profiles and then determine how these may relate to changes in various types of performance.

It has been hypothesized that for generating an effective stimulus it is fundamental to utilize an exercise mode that maintains a high mechanical power and therefore an elevated flux through a metabolic pathway in the working muscles [2,3]. Accordingly, protocols fitting this criteria, such as ~30-s “all-out” efforts interspersed by long rest periods (> 6 times the exercise duration)—named as speed endurance production (SEP) training [1], have shown to improve short-term supramaximal [6–11], medium- to long-term [6,7,9,12–18], repeated sprint [7,8,19,20] and exhaustive high-intensity intermittent [10] performances in both recreationally active and endurance-trained individuals. The notable efficacy of such training modality is likely related to adaptations in a number of key physiological variables including, among others, greater abundance and/or maximal activity of glycolytic [21,22] and mitochondrial oxidative [9,13,14,21–23] enzymes along with increased muscle content of pH regulatory [10,12] and K^+^ transporting [6,10] proteins.

On the contrary, other theories [2,11] sustain that rather than the rate of muscle contraction and metabolism, it’s the “chemical” environment inside the muscle cell, such as that created by protocols leading to a progressive accumulation of fatigue (e.g. high concentration of lactate, increased intracellular acidosis, or elevated levels of ions, inorganic phosphate and AMP), which plays a key role for promoting favourable physiological and performance adaptations. Consistently, in both untrained and aerobically-trained subjects, interventions encompassing work-to-rest ratios below 1:4 (speed endurance maintenance–SEM training) [1] have also invoked beneficial effects on the ability to perform repeated sprints [3], short- to long-term exercises [11,24,25] and high-intensity efforts to exhaustion in association with reduced rate of glycogen utilization [3], increased oxidative capacity [26,27], as well as improved pH regulation and ion homeostasis [3].

However, the changes in high-intensity exercise described in populations not accustomed to intermittent or multiple-sprint activities are not very elucidative for understanding how team sport athletes, such as football players, do respond to anaerobic training. Also, whilst it is well established that specifically designed speed or repeated sprint drills exert positive effects on performance-related fitness variables, in particular repeated sprint ability (RSA) [28–33], less evident are the advantages of speed endurance training on soccer-specific physical qualities. In particular, no information is available on athletic changes following a period of SEM training in soccer players, whereas only three studies [34–36] have investigated the impact of SEP training in soccer, but no clear consensus has been reached.

In the study by Thomassen et al. [35], a ~2% improvement in RSA with concomitant 14% higher amount of Na^+^,K^+^ pump α2 subunit was observed in sub-elite players undergoing a two-week post-season period consisting of five SEP and five aerobic high-intensity training sessions. Conversely, Gunnarsson et al. [36] noted moderate increases in the Yo-Yo Intermittent Recovery Test Level 2 (Yo-Yo IR2) performance (10.8%) and monocarboxylate transporter 1 (MCT1) protein expression (9%) after five SEP sessions, whereas junior players [34] showed greater results in the 10-m sprint (3.2%) and Yo-Yo IR2 (11.3%) but no changes in RSA, following six biweekly in-season SEP training sessions.

Unfortunately, comparing the effect of an intensified training protocol with a control period may not be very informative either, as the higher stimulus clearly leads to larger athletic improvements [37]. Thus, the aim of the present study was to compare the effect of speed endurance production and maintenance training on maximal and high-intensity intermittent exercise performance in soccer players. We hypothesized that SEP would improve the overall speed during repeated sprint exercise whereas SEM would increase the ability to tolerate fatigue development and sustain supramaximal efforts. A greater knowledge of how these training protocols affect different types of performance in individuals already familiar to high-intensity intermittent exercise, will extend the current understanding of the specificity of training adaptations.

## Methods

### Subjects

Eighteen young male outfield soccer players belonging to the same professional team and with a minimum of 8 yrs of experience were originally recruited to participate. Two of them were coming back from a long-term injury and were excluded *a priori*. Other three players got involved in first team duties and did not initiate the training intervention. As a result, 13 subjects (age 18.5 ± 1 yr, height 179.5 ± 6.5 cm, body mass 74.3 ± 6.5 kg) completed the study. The participants were actively involved in national level competitions and were fully informed of any possible risks and discomforts associated with the experimental procedures before giving their written informed consent to participate.

The study was conformed to the code of Ethics of the World Medical Association (Declaration of Helsinki) and was approved by the local Ethic Committee of Frederiksberg.

### Experimental Approach

In order to assess the changes in performance induced by two different regimes of speed endurance training, a parallel two-group, matched-work, longitudinal (Baseline test-Pre test-Post test) experimental design was used. The project was conducted during the last part of the competitive season (May) and consisted of a 2-wk baseline period followed by a 3-wk training intervention period.

To ensure that both groups presented equal pre-training average values for each performance variable, the participants were matched based on their initial (Pre test) athletic performances and randomly allocated to either a speed endurance production (SEP; n = 6) or speed endurance maintenance (SEM; n = 7) training group.

The performance tests were carried out at the beginning of the baseline as well as before and after the intervention period, and included: i) 20-, ii) 40- and iii) 200-m sprint, iv) a repeated sprint test (RSA) and v) the Yo-Yo Intermittent Recovery Test Level 2 (Yo-Yo IR2).

### Procedures

#### Training intervention

Before the intervention, players performed four training sessions and a full-length game per week. During the training intervention period, they conducted three weekly sessions (Monday, Wednesday, Friday) and one 45-min friendly match (Saturday).

As prior to the study, the duration of all training sessions was ~90 min and encompassed both warm up and technical/tactical skill development, with the exception of the last ~20 min where the habitual conditioning part (i.e. aerobic high-intensity, strength or speed/agility drills), was replaced by speed endurance training.

The SEP protocol consisted of 6–8 reps of 20-s all-out bouts interspersed with 120 s of passive recovery, whereas SEM training was characterized by 6–8 x 20-s all-out bouts followed by 40 s of passive recovery between repetitions. During each 20-s bout, players carried out single shuttle runs of approximately 140–150 m (70–75 + 70–75 m, depending on the group) at their full maximal effort, which were recorded by the use of portable photoelectric cells (Optojump, Microgate, Bolzano, Italy). In addition, session rate of perceived exertion (RPE) was collected ~20 min after every session using the Borg’s CR-10 scale and RPE-based training load was calculated as training duration x RPE score [38].

All SEM and SEP training sessions took place on artificial turf and were carefully supervised. During the first and the second session, the subjects underwent three and five repetitions, respectively. From the third, the players performed six to eight repetitions. During the 3-wk intervention period, the participants performed three speed endurance sessions per week (all had a compliancy of 100%). No other physical exercise was conducted aside from the one prescribed in the football environment. To minimize any potential interference of external variables, the players maintained their standard lifestyle and normal food intake in the weeks before and during the experiment.

#### Performance tests

Performance tests were carried out under ambient temperature of 22–27°C, and on three different experimental days following the same order during baseline and intervention period assessments. All players were familiarised with the testing procedures before the initial testing.

On the first experimental day, 20-, 40- and 200-m sprint tests were performed in the aforementioned order. Next, 24 h later the subjects underwent the RSA test. Finally, 48 h after, the Yo-Yo IR2 was performed.

On the days of testing, subjects reported to the pitch or track 2 h 30 min after having consumed a light meal. They also refrained from strenuous physical activity and abstained from alcohol and caffeine consumption 24 h before testing. To reduce the possible effect of diet-induced changes on muscle metabolism and subsequent exercise performance, two days before any experimental testing the participants were required to follow a nutritional strategy designed to ensure an adequate carbohydrate intake (~60% of total energy intake) and to record and replicate their individual dietary pattern during the 48-h before each testing day.

#### 20- and 40-m sprint tests

Short-sprint performance was evaluated over 20- and 40-m distances on artificial turf. Time was recorded using photoelectric cells (Optojump, Microgate, Bolzano, Italy). Players started the sprint from a standing position with the front foot placed 10 cm before the first timing gate. Only the best performance over three trials was considered. Each 20- and 40-m sprint was separated by 1.5 and 2.5 min of passive recovery, respectively, whereas a rest of 5 min was given between the two sets of sprint.

#### 200-m sprint test

A 200-m all-out run was performed on a 400-m athletics track 10 min after the 40-m sprint test. Time recordings were obtained as previously described for the short-sprint tests. Capillary blood samples were taken from the ear lobe at rest and 3 min after the 200-m sprint test. Blood lactate concentration ([La^-^]) was analyzed using a portable lactate analyzer (Lactate Plus, Nova Biomedical, Waltham, MA, USA).

Furthermore, the reliability of the 200-m sprint test was determined in all the thirteen subjects, who performed the tests during Baseline and Pre, and the test retest variability was calculated as the typical error expressed as a coefficient of variation. The coefficient of variation observed was 0.8 ± 0.7%.

#### RSA test

The RSA test was performed on artificial turf and it consisted of 15 repetitions of 40 m sprints interspersed with 30 s of passive recovery. This type of RSA with 15 reps has been commonly utilized in the literature [39,40]. Time was measured with photoelectric cells (Optojump, Microgate, Bolzano, Italy). Total sprint time (RSA_t_) for all 15 sprints was determined.

In addition, in order to quantify fatigue during the RSA test, the percentage decrement score (RSA_Sdec_) was calculated as follows [41]:

$$RSA_{Sdec}=\left[\frac{s_{1}+s_{2}+s_{3}+\ldots +s_{final}}{s_{best}\times number\;of\;sprints}-1\right]\times 100$$

The reliability of the RSA test was determined as reported for the 200-m sprint test. The coefficients of variation for RSA_t_ and RSA_Sdec_ were 1.2 ± 0.9% and 16.8 ± 14.9%, respectively, which are of similar magnitude as observed in previous studies [30,35].

#### Yo-Yo IR2 test

The Yo-Yo Intermittent Recovery Test level 2 was performed on artificial turf, after 15-min of standardized warm-up. The test consists of 2 x 20-m shuttle runs at increasing speeds, interspersed with 10 s of active recovery, controlled by audio signals. The test was terminated when the subject was no longer able to maintain the required speed and the distance achieved was recorded as result [42].

### Statistical Analyses

The normality distribution of each variable was examined with the Shapiro-Wilk test.

Data were analyzed using a two-factor repeated-measure ANOVA with one between factor (group: SEP vs. SEM) and one within factor (time: Pre vs. Post).

A two-factor repeated-measure ANOVA was also used to detect differences in the Pre test performance measurements between groups and to investigate within subjects differences between Baseline and Pre measurements.

When a significant main effect was detected, a Student-Newman-Keuls post hoc analysis was applied for pairwise multiple comparison.

In addition to the null-hypothesis test, to allow a better interpretation of the results, a statistical approach based on the magnitudes of change was also utilized to examine the practical significances [28,43,44].

For between- and within-group comparisons, the chances that the true mean changes following each training program were *beneficial* (i.e. greater than the smallest worthwhile change, SWC [0.2 multiplied by the between-subject standard deviation]), *unclear/trivial or harmful* for performance were calculated.

Quantitative chances of *beneficial*, *trivial*, or *harmful* changes were evaluated qualitatively as follows: <1%, almost certainly not; 1–5%, very unlikely; 5–25%, unlikely; 25–75%, possibly; 75–95%, likely; 95–99%, very likely; and >99%, almost certainly.

If the chances of having *beneficial* or *harmful* performance changes were both >5%, the true difference was defined as *unclear* [43,44]. Moreover, to calculate the effect size (ES) of changes in each performance parameter between SEP and SEM group, the pooled pre-training standard deviation was used [45]. Threshold values for Cohen ES statistic were >0.2 (small), 0.5 (moderate) and >0.8 (large).

The level of statistical significance was set for all analyses at p *<* 0.05. Raw data are presented as means ± SD, whereas relative changes are means ± 90% confidence intervals.

The relationship between relative changes in 200-m sprint performance and changes in rate of blood lactate accumulation was calculated using the Pearson’s product moment correlation coefficient (r).

The magnitude of the correlation was determined using the following criteria: r < 0.1, trivial; 0.1–0.3, small; 0.3–0.5, moderate; 0.5–0.7, large; 0.7–0.9, very large; 0.9–1.0 almost perfect.

Confidence intervals (CI) for correlations were calculated. If the 90% CI overlapped positive and negative values, the magnitude was considered to be unclear [44]. Statistical significance was set at p < 0.05.

## Results

### Training Load Measurements

**Fig 1:**
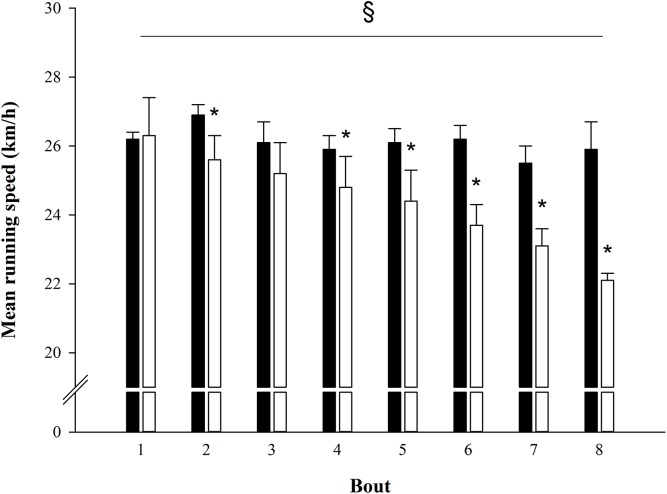
Mean running speed within 8 bouts of either a SEP (black bars) or a SEM (white bars) training session. * Significantly different from SEP (p < 0.05). § Significant “group x bout” interaction (p < 0.05)

A significant “group x bout” interaction (p < 0.001) was observed for the mean running speed maintained throughout the protocol (Fig 1). The speed decrement was greater during SEM (from 26.3 ± 1.1 to 22.1 ± 0.2 km/h) compared to SEP (from 26.2 ± 0.2 to 25.9 ± 0.8 km/h) training sessions. In addition, the running speed during the 2^nd^, 4^th^, 5^th^, 6^th^, 7^th^ and 8^th^ bout was lower in SEM than in SEP (p < 0.05).

No differences in the RPE-based training load (RPE-session) were detected between SEP and SEM in the nine training sessions performed during the intervention period (p > 0.05). Mean RPE-session was 620 ± 50 and 634 ± 45 AU in SEP and SEM, respectively.

### Performance

Performance measurements are reported in Table 1.

**Table 1:** Changes in performance following speed endurance production (SEP) and speed endurance maintenance (SEM) training.

	SEP (n = 6)		SEM (n = 7)		Changes observed for SEP compared with SEM			
	Pre	Post	Pre	Post	Standardized differences (Cohen’s d ± 90% CI)	Rating	Percent chances of better/trivial/worse effects	Qualitative inference
20 m (s)	2.84 ± 0.08	2.83 ± 0.12	2.91 ± 0.09	2.87 ± 0.10	-0.28 ± 0.68	Small	12/30/58	Unclear
40 m (s)	5.22 ± 0.09	5.22 ± 0.17	5.24 ± 0.11	5.20 ± 0.15	-0.24 ± 0.80	Small	17/29/53	Unclear
200 m (s)	25.95 ± 0.81	25.64 ± 0.99	26.59 ± 0.70	26.02 ± 0.62 *	-0.27 ± 0.50	Small	6/34/60	Unclear
Yo-Yo IR2 (m)	927 ± 185	1020 ± 155 * §	989 ± 226	1026 ± 210 *	0.28 ± 0.24	Small	74/26/0	Possibly
RSA_t_ (s)	86.09 ± 6.30	83.97 ± 4.72 * §	83.81 ± 2.37	84.65 ± 2.27	0.28 ± 0.32	Small	68/31/1	Possibly
RSA_Sdec_ (%)	5.03 ± 2.35	5.50 ± 2.98	4.07 ± 1.28	3.55 ± 1.01	-0.13 ± 0.75	Trivial	21/36/43	Unclear

* Significant difference from Pre (p < 0.05).

§ Significant “group x time” interaction (p < 0.05).

Pre intervention performance parameters were not different between the two groups. No differences were detected between Baseline and Pre intervention measurements in the fitness tests performed.

The Yo-Yo IR2 performance improved by 10.1% (p < 0.001) and 3.8% (p = 0.049) in SEP and SEM, respectively.

In SEP, RSA_t_ was 2.5% lower (p = 0.002) following the intervention, while no changes occurred in SEM (p = 0.1).

SEM improved the 200-m sprint time (-2.1%, p = 0.004), whereas only a tendency was observed in SEP (-1.2%, p = 0.087).

Significant “group x time” interactions were observed for Yo-Yo IR2 (p = 0.04) and RSA_t_ (p = 0.001).

No significant changes were observed in 20- and 40-m sprint time as well as in RSA_Sdec_ in neither SEP nor SEM.

### Magnitude-Based Approach—Within-Group Changes

**Fig 2:**
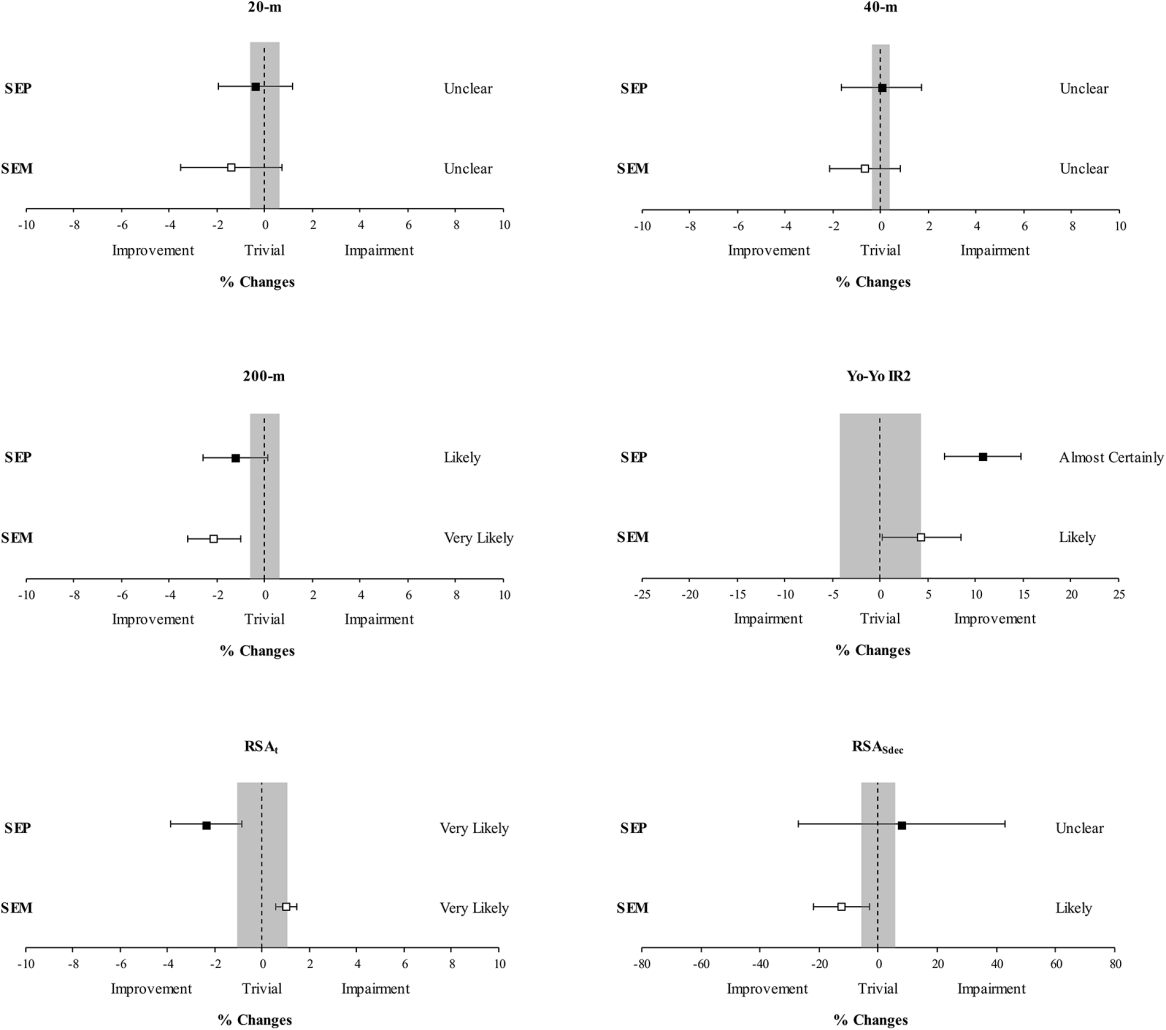
Relative changes for 20-m, 40-m and 200-m sprint time, Yo-Yo IR2 distance, total sprint time (RSA_t_) and percentage decrement score (RSA_Sdec_) of the repeated sprint ability test following speed endurance production (SEP) and speed endurance maintenance (SEM) training (bars indicate 90% confidence intervals). Trivial area was computed from the smallest worthwhile change (see methods).

The relative changes and qualitative inferences from within-groups analysis are outlined in Fig 2. Changes in 20-m sprint were *unclear* after both interventions, as chances that the true changes were *beneficial/unclear/harmful* were 78/15/7% and 45/40/15% following SEM and SEP, respectively. Similarly, also changes in 40-m sprint were *unclear* following both SEM (62/27/11%) and SEP (31/35/34%) training. Changes in 200-m sprint performance were *very likely* (99.0/0.7/0.3%) and *likely beneficial* (89/8/3%) in SEM and SEP, respectively. Improvements in Yo-Yo IR2 performance were *likely* (91/7/2%) and *almost certain* (99.8/0.1/0.1%) following SEM and SEP training, respectively. Changes in RSA_t_ were *very likely harmful* (0/3/97%) in SEM, while, on the contrary, *very likely beneficial* (97/2/1%) in SEP. Changes in RSA_Sdec_ were *likely beneficial* (88/11/1%) following SEM and *unclear* (55/23/22%) following SEP training.

#### Magnitude-based approach—Between-Group Changes

**Fig 3:**
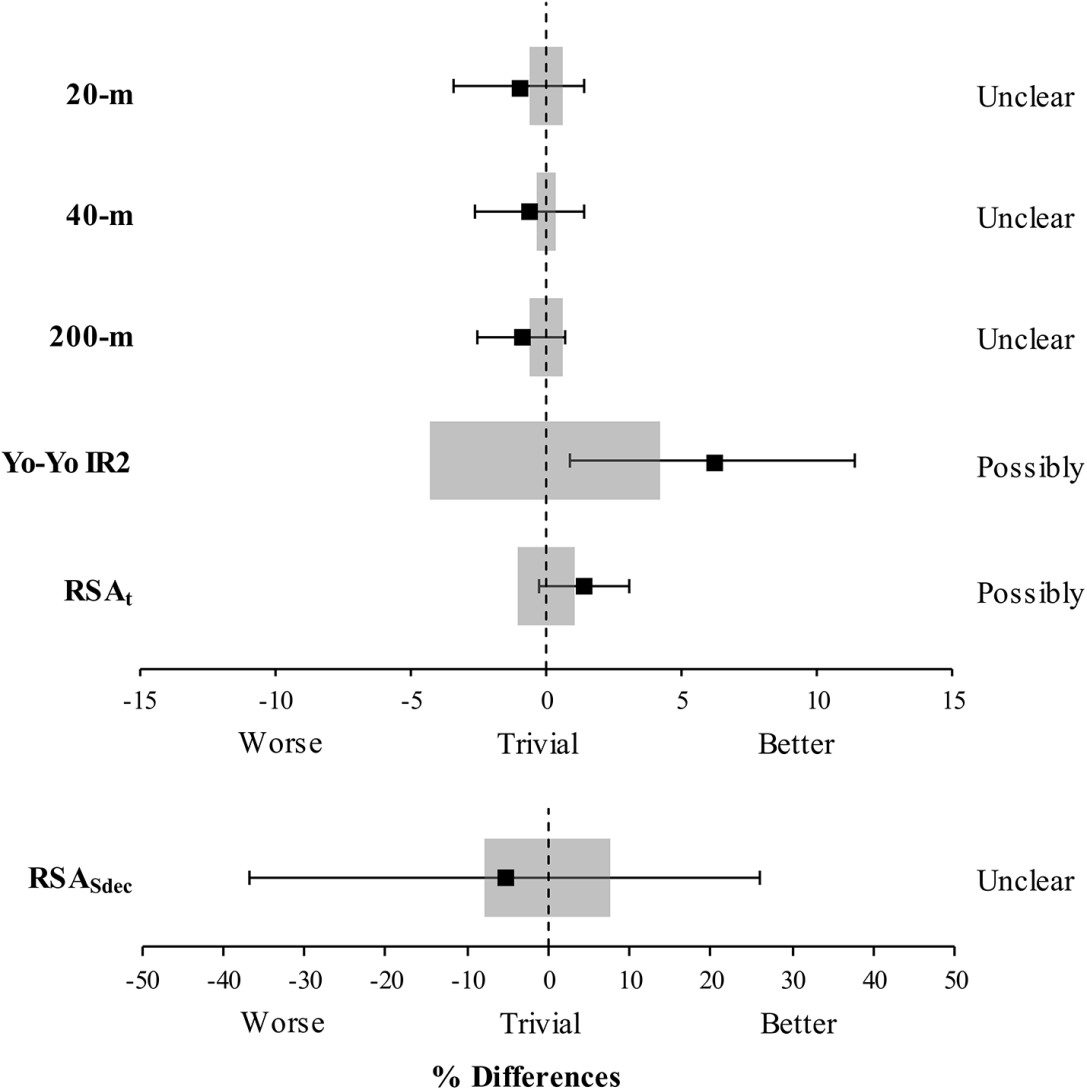
Speed endurance production (SEP) compared with speed endurance maintenance (SEM) training. Effectiveness of SEP compared with SEM training to improve 20-, 40- and 200-m sprint time, Yo-Yo IR2 performance as well as total sprint time (RSA_t_) and percentage decrement score (RSA_Sdec_) of the repeated sprint ability test.

Between-group changes are reported in Table 1 and presented in Fig 3. Changes in Yo-Yo IR2 and RSA_t_ were *possibily greater* in SEP than those observed in SEM. Differences in the changes of 20-m, 40-m, 200-m and RSA_Sdec_ were *unclear*.

### Blood lactate

Blood [La^-^] at rest was the same before and after the intervention period in both groups (p > 0.05).

Blood [La^-^] 3-min after the 200-m sprint test changed from 6.8 ± 1.7 to 7.8 ± 1.7 mmol/l (p < 0.05) and from 5.3 ± 0.7 to 6.6 ± 1.0 mmol/l (p < 0.01) following SEP and SEM, respectively.

No differences in blood [La^-^] were observed between SEP and SEM before and after the intervention period.

#### Relationship between rate of blood lactate accumulation and 200-m sprint performance

**Fig 4:**
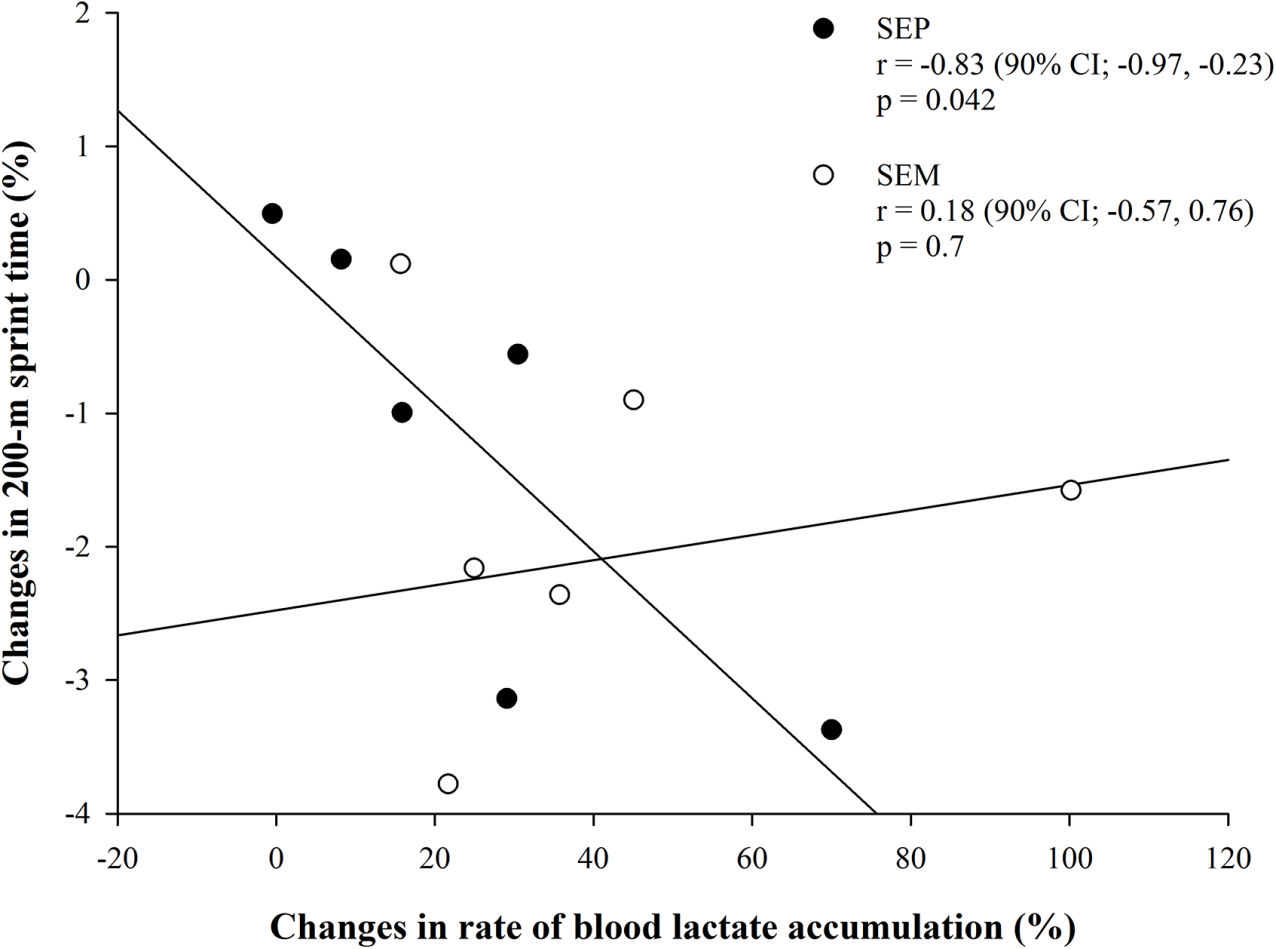
Relationship between relative changes in 200-m sprint time and changes in rate of blood lactate accumulation.

In SEP, the improvement in 200-m sprint performance was correlated with the increase in rate of blood lactate accumulation (r = -0.83; p = 0.042; Fig 4), while no relationship was observed in SEM (r = 0.18; p = 0.7).

## Discussion

The main findings of the current study were that SEP training lowered the total time in the repeated sprint test (RSA_t_) and showed to be *likely* effective for enhancing the 200-m sprint performance. SEM training improved the 200-m sprint performance and had a *likely* beneficial impact on the percentage decrement score (RSA_Sdec_) of the repeated sprint test but induced a *very likely* impairment in RSA_t_. Both training interventions conferred positive changes in the Yo-Yo IR2, with *possibly* greater improvements following SEP compared to SEM. No differences were observed in the 20- and 40-m sprint performances.

To our knowledge, the present investigation is the first to examine the impact of SEM training on footballers’ ability to perform repetitive maximal and high-intensity exercise as well as to compare the effectiveness of two distinct anaerobic training approaches (SEP and SEM) on a variety of soccer-related fitness tests.

### Repeated Sprint Ability

Performance in RSA_t_ was improved following SEP whereas it was *very likely* impaired following SEM. Speed endurance production encompassed running bouts carried out at maximal, near maximal speed whereas SEM training resulted in a gradual decrease of exercise intensity as the training progressed (Fig 1). This indicates that, in people habitually used to intermittent exercise, generating elevated power output and maintaining very high speed throughout the entire protocol is key for improving the ability to perform repeated sprints or all-out efforts. Apparently, in order to sustain maximal performance in each subsequent exercise bout, the recovery periods are required to be sufficiently long (for the muscles to restore or get close to their pre-exercise status), whereas the work duration should be relatively short but at the same time long enough for optimally taxing the glycolytic energy system [1,16,46,47]. The present findings would explain why studies employing SEM-oriented combinations (lower work-rest ratio, i.e. 30 s exercise and 2 min rest) [29] or higher volumes of long (i.e. 40 s) speed endurance runs [34], failed to attain positive changes in the mean sprint time during a RSA test in trained junior handball and soccer players, respectively.

Conversely, in line with the current observation, improvements in the mean repeated sprint time was reported in a group of soccer players who for a two-wk period underwent five aerobic high-intensity and five soccer-specific SEP (incorporating 25-s bouts separated by 3 min of rest) training sessions [35]. Moreover, the magnitude of the improvements in the present study are comparable to those achieved by team sport players after both sprint [32] and repeated sprint [28–30] training.

Thus, in accordance with previous investigations [29], it appears legitimate to affirm that, in team sports athletes, “maximization of RSA_t_ is linked to the ability of developing maximal speed”, which, as demonstrated in the current study, can also be fully achieved by proper manipulation of SEP training regimes.

Conversely this is not necessarily the case for physically active [3,48] and endurance-phenotype [49] subjects in whom reduced RSA_t_ or increased RSA total work was noticed also in response to SEM-based training. Apparently, from a transcriptional point of view, also molecular intermediates derived from the metabolic processes not involving maximal rate of muscle contraction, may act as dominant triggering signals for promoting optimal adaptation in the capacity to produce and reiterate powerful burst continuously.

Taken together, these results suggest that, depending on the population and its fitness status, equivalent athletic improvements can be induced also via weaker speed-related stimuli, and may consequentely rely on different physiological mechanisms as demonstrated by previous studies where untrained [9,12], endurance runners [6,10] and soccer players [35,36] showed similar SEP training-induced performance changes in association with different muscle adaptations.

When examining the changes in RSA_Sdec_, practically *likely* improvements were displayed only following SEM training. This current trend complies with previous observations showing beneficial changes in RSA_Sdec_ after different SEM-oriented training regimes [3,29]. Thus, in contrast to what was noted in RSA_t_, when the aim is improving fatigue resistance, training interventions characterized by short recovery periods which elicit pronounced metabolic disturbances [11] and perturbations in ion homeostasis [3], would seem to be superior for minimizing the decline in muscle force/speed production during repeated sprint activities.

These results show that SEP and SEM training strategies target different components of RSA [47] and confirm that for optimizing RSA it is preferable/advantageous to stress each of the limiting mechanisms in isolation rather than soliciting the multiple facets of RSA performance concurrently [5].

### 200-m Sprint

The observation that the 200-m sprint time was significantly reduced only after SEM, indicates that training modes leading to a progressive reduction in power output and simultaneous fatigue development as the exercise continues, provide stronger stimuli for improving the ability of a muscle to preserve speed decrement and sustain short-duration supra-maximal/all-out exercise. Consistently, greater effects in improving speed maintenance during runs of 200 and 300 m were shown when 10 s repeated sprints were carried out with a 1:1 rather than a 1:6 exercise-to-rest-ratio [11]. Shorter rest intervals may indeed limit the resynthesis of phosphocreatine (CP) with the result of lowered net CP availability and increased degradation products (i.e. Pi, AMP, ADP) at the onset of each subsequent bout [50]. This would in turn tax glycolysis to a greater extent [51], as demonstrated by larger amount of glycolytic intermediates (i.e. glucose-6-phosphate, fructose-6-phosphate), and lead to tangible performance advantages in terms of speed maintenance [11].

Among other factors, the improvement in 200-m sprint time could also be ascribed to changes in capillary density as this was calculated to account for 50% of the variance observed in exercise lasting 30 s [52], and SEM [53] but not SEP [36,54] has been shown to increase capillarization. However, no information is available about the impact of SEM training on endothelial cells proliferation in trained populations and thereafter it is still unclear whether the improved angiogenesis denoted is a direct effect of the SEM exercise protocol per se or it is more a consequence of the fact that the study by Jensen et al. [53] involved untrained individuals.

On the other hand, a practical *likely* beneficial effect on 200-m performance was also noticed in SEP, suggesting that besides fatigue tolerance, also biochemistry processes implicated in producing elevated power outputs (counting also FTx distribution) [52], could be of importance for generating and maintaining maximal speeds over relatively prolonged intervals of time (e.g. 30 s). This notion is strengthen by the finding that reduced sprint time over 200 m was associated in both groups to higher blood lactate accumulation, but the percentage of performance improvement in SEP and not in SEM, was related to the relative rise in blood La^-^ accumulation, likely reflecting an augmented ability in ATP production via the anaerobic glycolytic pathway following SEP training. Thus, a high rate of anaerobic energy release may also have been part of the explanation for the improved RSA_t_. Also a mitigated drop in muscle ATP through an increase in ATP resynthesis has been suggested as another plausible explanation for the observed improvements [11].

Hence, although at first glance long duration all-out bouts may seem of limited relevance for typical football activity profile [55], recent data (unpublished observation) have proved that supramaximal long efforts (e.g. vigorous box-to-box runs) are clearly present during the intense phases of a football game, and thereafter certain players may benefit from having a well-equipped ability to perform more prolonged power running.

### High-Intensity Intermittent Exercise Capacity

Both interventions induced improvements in the Yo-Yo IR2 test, denoting that the typical traits of each training (i.e high speed development and fatigue resistance) are essential for optimally performing high- to-“all-out” intensity bouts repeatedly. However, given that the observed effect of SEP on Yo-Yo IR2 was practically *almost certain* and *possibly* greater compared to SEM (~10 vs ~4%, respectively), the primary mechanisms underlying Yo-Yo-type 2 performance improvements, would seem to withstand chiefly on the muscle’s ability to produce forceful bouts rather than to increase exercise tolerance. The pronounced development of fatigue during SEM may have indeed limited the recruitment of some muscle fibers resulting in reduced pool of fibers available for adaptations.

Despite limited speed endurance exposure (only nine sessions) and overall ~20% reduction in total volume, the current SEP-induced changes in Yo-Yo IR2 performance are of similar magnitude of those previously found in trained soccer players [34,36] and runners [10] (~11 and 19%, respectively), but remarkably different (3 fold lower) from what observed after a SEM period in recreationally active subjects [3]. Apparently, SEM training effects on intermittent exhaustive performance appear to be drastically different depending on the population recruited, though it should be taken into account that, compared to the present, in the study by Mohr et al. [3] the amount of speed endurance training was ~3 times bigger.

The fact that the peak and mean heart rate responses (and likely also tissue blood flow, O_2_ delivery and VO_2_) were shown to be lower during SEP compared to SEM training [56], indicates that repeated all-out efforts and power-related adaptation, vital in the contemporary football game [57], are not strongly cardiovascular-related and improvements in high-intensity performance may occur in spite of weaker overload of the oxygen-dependent system. Concordantly, in trained humans, the SEP-induced improvements in a variety of very intense exercise forms were shown to be associated primarily with peripheral remodeling including restructure of muscle architecture [8,36,54], changes in membrane transport proteins involved in regulating muscle pH [10,36] and preserving cell excitability [6,10,35,36], but not with VO_2_max [6,10,35,36,54] or mitochondrial enzymes [6,36,54,58] enhancement.

### 20- and 40-m Sprints

Consistently with the vast majority of studies [3,36,59], no effects of speed endurance training were noticed on single short-sprint performances (i.e. 20 and 40 m), indicating that the impact of this form of conditioning on neural aspects and purely speed-related components is minimal also in team sports individuals.

## Conclusion

In conclusion, SEP training comprising relatively brief work bouts and fairly long recovery periods, is a potent stimulus to substantially improve high-intensity intermittent exercise performance and the overall speed during repeated sprint efforts in trained soccer players. Conversely, SEM training, characterized by shorter rest intervals, reduces power decrement and maintaines speed development during both repeated all-out and continuous short-term maximal exercises.

Thus, speed endurance training undoubtedly has usefulness in ameliorating the physiological mechanisms that limit soccer-related exercise performance.

Furthermore, despite the limited number of subjects, the differences between the two protocols are evident and clearly indicate that adaptations are specific to the training characteristics, providing new insight into the nature of a stimulus necessary to improve specific types of athletic performance in trained team-sports individuals.

### Practical Applications

These two training regimes target different determinants of physical performance. Therefore, in team sport settings, it is paramount that exercise training is appropriate for the intended physiological and performance adaptations (e.g. generating maximal runs vs developing fatigue resistance). The selection of which approach to prioritize should be based on the players’ characteristics and individual game requirements.

### Perspectives

Future studies are warranted to investigate the acute metabolic and molecular responses along with long-term muscular adaptations, which underpin the divergent changes observed in athletic performance following SEP and SEM.
